# iASeq: integrating multiple chip-seq datasets for detecting allele-specific binding

**DOI:** 10.1186/1471-2105-13-S18-A6

**Published:** 2012-12-14

**Authors:** Yingying Wei, Xia Li, Qianfei Wang, Hongkai Ji

**Affiliations:** 1 Johns Hopkins University, Baltimore, Maryland, USA; 2 Beijing Institute of Genomics, Chinese Academy of Sciences, No.7 Beitucheng West Road, Chaoyang District, Beijing 100029, PR China

## Background

ChIP-seq provides new opportunities to study allele-specific protein-DNA binding (ASB). Detecting ASB from ChIP-seq often suffers from low statistical power since only sequence reads mapped to heterozygote SNPs are informative for allelic inference. Moreover, little is known about the correlation patterns of ASB among different transcription factors and histone modifications.

## Methods

We address both issues by developing iASeq to jointly analyze multiple ChIP-seq datasets. iASeq uses a Bayesian hierarchical model to automatically discover correlation patterns of ASB among multiple proteins. It then uses this correlation to borrow information across datasets to improve ASB detection.

Application of iASeq to 57 ENCODE datasets consisting of 117 ChIP-seq samples from GM12878 cells discovers synergistic allele-specificity patterns across different proteins. The analysis also shows the ability of iASeq to increase the power of ASB detection compared with the traditional approach which analyzes each individual dataset separately. According to two gold standards, iASeq's increased the accuracy of identifying ASB substantially.

## Conclusions

iASeq demonstrates the value of integrating multiple datasets in the analysis of ASB, and it offers a new tool to better characterize allele-specificity. A user friendly R package is made available for iASeq. In principle, the same model can be applied to call allele-specific expression and allele-specific methylation by integrating multiple RNA-seq and MeDIP-seq studies.

